# EGF regulates survivin stability through the Raf-1/ERK pathway in insulin-secreting pancreatic β-cells

**DOI:** 10.1186/1471-2199-11-66

**Published:** 2010-08-31

**Authors:** Haijuan Wang, Katarina Gambosova, Zachary A Cooper, Michael P Holloway, Andrea Kassai, Denisse Izquierdo, Kelly Cleveland, Charlotte M Boney, Rachel A Altura

**Affiliations:** 1 Department of Pediatrics, Division of Pediatric Hematology-Oncology, Brown University, Providence, RI, 02903 USA; 2 Department of Pediatrics, Division of Pediatric Endocrinology, Brown University, Providence, RI, 02903 USA

## Abstract

**Background:**

Postnatal expansion of the pancreatic β-cell mass is required to maintain glucose homeostasis immediately after birth. This β-cell expansion is regulated by multiple growth factors, including glucose, insulin, insulin-like growth factor (IGF-1) and epidermal growth factor (EGF). These mitogens signal through several downstream pathways (AKT, ERK, STAT3, and JNK) to regulate the survival and proliferation of β-cells. Survivin, an oncofetal protein with both pro-proliferative and anti-apoptotic properties, is a known transcriptional target of both IGF-1 and EGF in cancer cells. Here, we analyzed the effects of the β-cell mitogens IGF-1 and EGF on survivin regulation in the established pancreatic β-cell model cell lines, MIN6 and INS-1 and in primary mouse islets.

**Results:**

In pancreatic β-cells, treatment with glucose, insulin, or EGF increased survivin protein levels at early time points. By contrast, no significant effects on survivin were observed following IGF-1 treatment. EGF-stimulated increases in survivin protein were abrogated in the presence of downstream inhibitors of the Raf-1/MEK/ERK pathway. EGF had no significant effect on *survivin *transcription however it prolonged the half-life of the survivin protein and stabilized survivin protein levels by inhibiting surviving ubiquitination.

**Conclusions:**

This study defines a novel mechanism of survivin regulation by EGF through the Raf-1/MEK/ERK pathway in pancreatic β-cells, via prolongation of survivin protein half-life and inhibition of the ubiquitin-mediated proteasomal degradation pathway. This mechanism may be important for regulating β-cell expansion after birth.

## Background

Production and maintenance of the pancreatic β-cell mass is a highly regulated process driven by four major mechanisms that include- β-cell replication, β-cell neogenesis, β-cell hypertrophy and β-cell apoptosis [1,2]. In the rodent, an exponential expansion of the pancreatic β-cell mass begins during the final phase of gestation and lasts through the third week after birth. Correspondingly, in humans, β-cell expansion occurs during the last trimester of pregnancy and continues through the first few months of life [1,2]. An increase in β-cell mass is required for insulin secretion in the maintenance of metabolic homeostasis [3], both in the initial transition to a carbohydrate-based diet following weaning and throughout life thereafter [4]. The molecular mechanisms regulating β-cell growth are mostly unknown but are dependent on a variety of growth factors, including glucose, insulin, insulin-like growth factor (IGF-I), and epidermal growth factor (EGF) [5,6], that provide mitogenic signals to the β-cell *in vivo*.

Epidermal growth factor receptor (EGFR) is a member of the ErbB receptor family, consisting of 4 transmembrane tyrosine kinase receptors: EGFR (ErbB1, HER1), ErB2 (neu/HER2), ErbB3 (HER3) and ErbB4 (HER4) [7,8]. All such proteins contain an extracellular domain responsible for ligand binding, a single membrane-spanning domain, and a cytoplasmic tyrosine kinase domain with multiple auto-phosphorylation sites. Binding of a ligand to EGFR leads to the formation of homo- or heterodimers, followed by phosphorylation of tyrosine residues and second messenger recruitment [7,8]. EGF is a potent growth factor and one of the 11 ligands of this receptor that signals via multiple downstream pathways including: PI3K/AKT, ERK1/2, JNK, JAK/STAT3, and others, dependent on which of the 5 tyrosine residues is phosphorylated [7].

EGFR signaling is critical for pancreatic development and for β-cell proliferation, as shown by *EGFR *knock-out and transgenic mouse models. Genetic disruption of *EGFR *is lethal in the embryonic and peri-embryonic periods and the pancreatic phenotype reveals a reduced pancreas size due to impaired ductal branching, abnormal islet cell localization, and defective differentiation [9-12]. Embryonic cell cultures established from these mice show a 50% reduction of β-cell mass, without impairment of other islet cell types [9]. After birth, tissue-specific attenuation of EGFR signaling in the β-cell using a dominant negative EGFR (EGFR-DN) that lacks 40% of tyrosine kinase activity leads to a failure of postnatal β-cell proliferation and islet mass expansion, resulting in insulin-deficient diabetes by two weeks of life [13]. This suggests that EGFR signaling after birth is critical for β-cell proliferation.

Survivin is the smallest member of a well-conserved protein family known as inhibitor of apoptosis proteins (IAPs) [14]. In cancer cells, survivin has at least two established functions; one as an inhibitor of programmed cell death [15] and the other as a regulator of cell division [16]. To perform its diverse functions, the survivin protein must shuttle between multiple subcellular compartments, including the cytoplasm, mitochondria, and nucleus [17]. Evidence suggests that survivin can inhibit both the extrinsic and intrinsic (mitochondrial) pathways of programmed cell death by blocking the activity of several caspase proteins [18,19]. Survivin also forms a complex with a group of chromosomal passenger proteins including Aurora B kinase, INCENP, and Borealin [20,21] to regulate cell division. Phosphorylation of survivin at threonine 117 by Aurora-B regulates survivin-targeting to the centromere and thus the entire chromosomal passenger complex [22,23]. Phosphorylation at an additional site, threonine 34, is critical for the anti-apoptotic function of survivin; whereby mutation at this site results in caspase 3 activation and mitochondrial apoptosis [24].

During embryogenesis (E15.5) in the mouse, survivin is expressed throughout the pancreatic epithelium [25]. Around the end of gestation, it becomes gradually restricted to endocrine cells. Postnatally, its expression becomes further restricted, where eventually (P21) it is expressed exclusively within the pancreatic β-cells [25]. In previous work, we engineered mice harboring a conditional deletion of *survivin *in pancreatic endocrine cells by mating *survivin *floxed mice with mice expressing a Cre-recombinase protein under the control of a *Pax-6 *promoter [25]. These mice developed insulin-deficient diabetes after birth due to a failure of β-cell mass expansion [25]. On a cellular level, we observed a slowing of cell cycle progression through G1/S and G2/M in the *survivin *null β-cells, which correlated with an increase in expression of the cell cycle inhibitor, *p21^WAF1^*. Similar findings were also observed in a *Pdx-1Cre;survivin*^*lox/lox *^mouse model [26]. In other related work, transplantation of pancreatic β-cells engineered to ectopically express survivin from a rat-insulin promoter into streptozotocin-treated mice resulted in long-term correction of hyperglycemia and rescue of streptozotocin-induced β-cell death [27]. Together, these data suggest that survivin is important in both the normal expansion of the β-cell mass after birth and in the survival of β-cells following stress-induced apoptosis.

As both EGF and survivin are essential for β-cell proliferation, and as survivin expression is regulated by EGF in cancer cells, we hypothesized that EGF also regulates survivin expression in β-cells and thereby is one of the mechanisms involved in promoting β-cell growth. We chose the well-established insulin-producing β-cell lines, MIN6 and INS-1, as an experimental model system to test this hypothesis. Here, we show that survivin is regulated by several pancreatic β-cell growth factors, including glucose, insulin, and EGF. Induction of survivin by EGF occurs extremely quickly, within 15 minutes of treatment. The mechanism of EGF-induced survivin occurs primarily through activation of the ERK pathway and prolongation of survivin half-life by inhibiting ubiquitin conjugation on the survivin protein. Thus, we have identified a novel mechanism for survivin regulation in pancreatic β-cells that implicates ERK as a critical molecule for its post-translational modification and signaling for protein degradation.

## Results

### EGF regulates survivin protein expression in pancreatic β-cells

**Figure 1:**
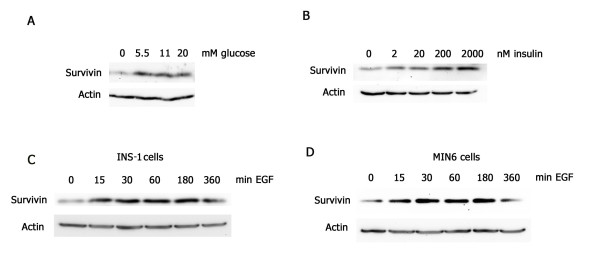
**Glucose, insulin, and EGF induce survivin protein expression**. MIN6 and INS-1 cells were serum-deprived for 2 to 4 hours (A and B) or overnight (C and D) then glucose, insulin, or EGF (50 ng/ml) were added at the indicated concentrations and times. (A) Survivin protein levels in response to glucose in MIN6 cells. (B) Survivin protein levels in response to insulin in MIN6 cells. (C) Survivin protein levels in response to EGF in INS-1 cells. (D) Survivin protein levels in response to EGF in MIN6 cells.

To begin to understand the mitogenic responsiveness of survivin in pancreatic β-cells we made use of the immortalized mouse and rat β-cell lines, MIN6 and INS-1. MIN6 cells were cultured under proliferating conditions then serum-and glucose-deprived for 2 to 4 hours, prior to treatment for 30 minutes with glucose or insulin. Results showed that varying concentrations of glucose (0 to 20 mM) or insulin (0 to 2000 nM) added to the cells can induce survivin protein expression at these early time points. MIN6 cells treated with glucose had a 10-fold increase in survivin protein levels at a concentration of ≥ 5.5 mM (Fig. 1A). Cells treated with insulin at a concentration of ≥ 20 nM had a similar increase in survivin levels (Fig. 1B). As IGF-1 and EGF are both known to stimulate survivin in cancer cells [28,29], we next tested whether these growth factors can also induce survivin in pancreatic β-cells. MIN6 and INS-1 cells were serum-deprived overnight then treated with IGF-1 (100 ng/ml) or EGF (50 ng/ml) for serial time points (0 to 360 minutes). EGF-treated cells showed a five- to ten-fold increase in survivin protein levels within 15 to 30 minutes after treatment (Figs. 1C and 1D), with no differences observed following IGF-1 treatment (not shown). These early increases in protein expression suggested that EGF likely regulates molecular mechanisms that modify survivin protein stability, rather than its transcription or translation.

**Figure 2:**
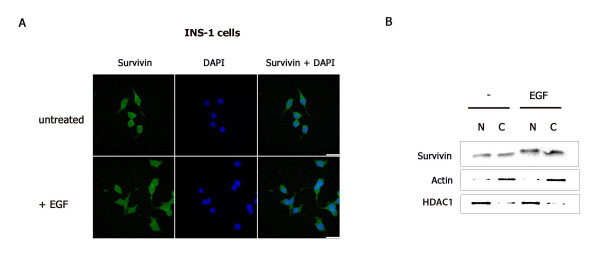
**EGF enhances nuclear and cytoplasmic survivin protein levels**. INS-1 cells were serum-deprived overnight then treated with or without EGF for 4 hours. (A) Cells were fixed and immunostained with a survivin antibody, stained with DAPI (DNA detection) then analyzed by Z-stack confocal microscopy. (B) Nuclear and cytoplasmic protein fractions were prepared, resolved by SDS-PAGE then transferred to PVDF membranes and immunoblotted with anti-survivin, anti-actin, and anti-HDAC1.

As survivin is a multifunctional protein whose diverse activities are carried out in different subcellular compartments [30], we next sought to gain insight into the potential effects of EGF on survivin localization. To this end, we performed indirect immunofluorescence staining using a survivin antibody in the presence or absence of EGF, to visualize the endogenous survivin protein within INS-1 cells, along with cell fractionation to quantify survivin expression within these compartments. Survivin localized to both nuclear and cytoplasmic compartments of untreated and EGF-treated cells, as observed by indirect immunofluorescence (Fig. 2A). An increase in both nuclear and cytoplasmic survivin protein was observed by cell fractionation following EGF treatment (Fig. 2B). These results suggest that EGF stimulates both the anti-apoptotic and pro-mitotic functions of survivin in pancreatic β-cells.

### EGF regulates survivin through ERK activation

**Figure 3:**
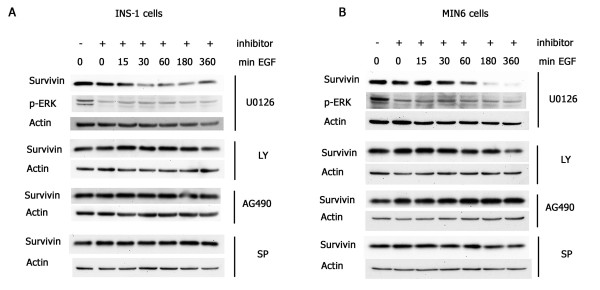
**EGF induces survivin through ERK activation**. INS-1 (A) and MIN6 (B) cells were serum-deprived overnight then treated with inhibitors to ERK1/2 (U0126), PI3/AKT (LY294002), STAT3 (AG490), and JNK (SP600125) for 30 minutes prior to the addition of EGF. Proteins were harvested at the indicated times for Western blot analysis and immunoblotted with anti-survivin, anti-actin, and anti-phosphorylated-ERK (p-ERK).

EGF signals through several downstream signaling pathways to induce β-cell growth, including PI3K/AKT, ERK1/2, JNK and JAK2 [7,8]. To determine which of these pathways might be required for EGF-stimulated survivin protein expression, we treated MIN6 and INS-1 cells with specific inhibitors of these pathways including: LY 294002 (PI3K/AKT), UO126 (ERK1/2), SP600125 (JNK) and AG490 (JAK/STAT3). The EGF-stimulated increase in survivin protein was only abrogated in the presence of the ERK1/2 inhibitor (Fig. 3A and 3B), suggesting that the ERK pathway is the primary signaling pathway involved in EGF-mediated survivin regulation. Interestingly, the response to the ERK inhibitor U0126 differed in the two cell lines. U0126 treatment of INS-1 cells blocked survivin induction within 15 to 30 minutes, while treatment of MIN6 cells blocked survivin induction at later times (30 to 60 minutes) with a further decrease in survivin observed at 180-360 minutes in these cells. This suggests that there is a cell or species-specific kinetics regulating the ERK-mediated increase in survivin levels, in response to EGF.

### EGF inhibits survivin protein degradation

**Figure 4:**
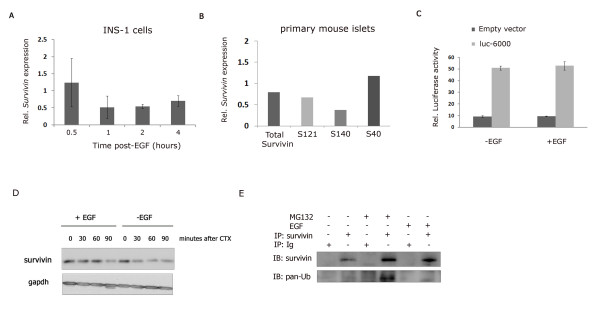
**EGF inhibits survivin protein degradation by blocking survivin ubiquitination**. (A) INS-1 cells were starved overnight then treated with or without EGF for serial time points, as indicated. *Survivin *mRNA levels in the presence of EGF were compared to those in the absence of EGF, as performed by quantitative RT-PCR (qRT-PCR). The relative levels and standard error (SE) of triplicate experiments are shown. (B) Primary mouse islets were starved overnight then treated with or without EGF for 2 hours. qRT-PCR was performed for all the mouse *survivin *splice forms (*survivin *121, 140, and 40). Bars represent the relative levels of duplicate experiments. (C) MIN6 cells were transfected with a *survivin *promoter construct regulating luciferase (luc-6000) or control plasmid (EV). Reporter activity was measured after overnight serum-deprivation and 2 hour EGF treatment. The mean and SE of triplicate experiments is shown. (D) INS-1 cells were starved overnight then treated with EGF for 4 hours. CTX (100 mg/ml) was added at the indicated times prior to the completion of the EGF treatment and protein harvested for Western blot analysis. (E) INS-1 cells were starved overnight then treated with the proteasome inhibitor MG132 for 3 hours followed by EGF for 1 hour. Lysates were harvested and immunoprecipitated with anti-survivin or immunoglobulin (Ig) control, resolved by SDS-PAGE, transferred to PVDF membranes and immunoblotted with anti-survivin or anti-ubiquitin (pan-Ub).

To determine the biochemical mechanism of the observed early increase in survivin protein levels following EGF treatment in pancreatic β-cells, we first evaluated potential changes in *survivin *mRNA and *survivin *promoter activity. Serum-deprived INS-1 cells were treated with EGF (100 ng/ml) for 0.5, 1, 2, and 4 hours prior to harvesting RNA for quantitative RT-PCR. No significant changes in *survivin *mRNA were observed at any of these time points (Fig. 4A), suggesting that EGF does not likely regulate *survivin *transcription in INS-1 cells. To validate this finding in primary islet cells, we isolated islets from 8-week old C57/Bl6 mice, serum-starved them overnight then incubated them with EGF (100 ng/ml) for 2 hours. Following mRNA preparation, we performed quantitative RT-PCR using primers to detect total mouse *survivin *and three mouse *survivin *splice forms, *survivin *121, *survivin *140 and *survivin *40 [31]. No significant increases in *survivin *mRNA following EGF treatment were observed in the primary islets (Fig. 4B), similar to the results obtained in the INS-1 cells. To examine whether this observation correlated with a lack of stimulation of *survivin *promoter activity, we transfected MIN6 cells with luciferase reporter constructs containing different regions of the *survivin *promoter, from 400 to 6000 bp upstream of the *survivin *ATG start site [32]. Twenty-four hours after transfection, cells were serum-deprived overnight then treated with EGF or vehicle control for 2 hours. No significant differences in reporter levels between EGF-treated and untreated cells were observed (Fig. 4C and data not shown), indicating an absence of EGF-stimulated transcriptional regulation sites within the *survivin *promoter.

As no significant increases in *survivin *mRNA or its promoter activity were observed following EGF treatment, we hypothesized that EGF was acting at a post-transcriptional level to either increase ribosomal-mediated protein synthesis or to inhibit protein degradation. To test these possibilities, we treated INS-1 cells with the protein synthesis inhibitor, cyclohexamide (CTX). Cells were serum-deprived overnight then treated with EGF. CTX was added for 0 to 90 minutes prior to cell collection at four hours post EGF treatment. In control cells, a decrease in survivin protein levels was observed within 30 minutes, in agreement with published results of survivin protein half-life [33] however, in EGF-treated cells no significant decrease in protein levels was observed until 90 minutes after CTX treatment (Fig. 4D). This supports the hypothesis that EGF can inhibit survivin degradation to prolong the survivin half-life.

Degradation of survivin protein normally occurs through activation of the ubiquitin-proteasome pathway [34]. To investigate whether EGF might protect survivin from degradation by inhibiting this pathway, we treated INS-1 cells with the 26 S proteasome inhibitor, MG132 [35]. Lysates were immunoprecipitated with a survivin antibody or immunoglobulin control antibody then immunoblotted with anti-survivin or anti-ubiquitin. Results showed an increase in total survivin protein levels, both in the presence of the proteasome inhibitor and following EGF treatment (Fig. 4E). Treatment with MG132 also led to an accumulation of the ubiquitinated form of survivin, as expected from the results of prior studies showing that survivin is degraded via this pathway [33,34]. By contrast, EGF treatment resulted in a decrease in ubiquitinated-survivin (Fig. 4E), suggesting that EGF inhibits conjugation of ubiquitin on the survivin protein, prior to proteolytic activation of the 26 S proteasome.

## Discussion and Conclusions

During late embryogenesis and immediately after birth, a transient burst of replication of pancreatic β-cells occurs with a consequent marked increase in β-cell growth [1,2]. New β-cells continue to form in the adult animal as well, primarily from the replication of mature β-cells [36]. This replication mechanism also appears to be of primary importance in humans, with the highest rates occurring within the first year of life [37]. Our previous data showed that mice lacking *survivin *within pancreatic β-cells develop insulin-deficient diabetes and are unable to expand their β-cell mass after birth. This phenomenon is due primarily to an inhibition of cell cycle progression [25], suggesting that the predominant effect of survivin in β-cells is to induce postnatal proliferation. Here, we aimed to examine the potential factors upstream of survivin that might regulate survivin expression in β-cells. As EGF-ligand activation of the EGFR receptor is important for maintaining β-cell mass as well as for β-cell function [8], and as it is known to regulate *survivin *transcription in cancer cells [29], we hypothesized that it might also regulate survivin in β-cells during periods of active replication. In this report, we demonstrate that survivin protein expression is enhanced at early time points following treatment with critical β-cell growth factors including glucose, insulin, and EGF. The molecular mechanism of the EGF-mediated increase in protein expression primarily involves activation of the ERK signaling pathway.

EGFR signaling leads to the activation of several downstream cascades, with the two major pathways including PI3-kinase/AKT and Ras/Raf-1/ERK [38]. Although much of the work studying these pathways has been performed in cancer cells, it has recently been demonstrated that endogenous Raf-1 signaling is required to suppress basal β-cell apoptosis [39] and that Raf-1 also participates in β-cell proliferation [40,41]. Further, low concentrations of insulin (0.2-20 nM) rescued human and rodent islets from serum withdrawal-induced apoptosis through Raf-1 activation [42,43] and islets isolated from mice with a 50% reduction in glucose-stimulated insulin secretion had a 50% loss of ERK activation in response to glucose [44]. Glucose and insulin stimulate nuclear translocation of ERK, an event that has been proposed to promote β-cell survival and growth via ERK-dependent transcription [44]. Our finding that EGF-mediated signaling through the Raf-1/ERK pathway prolongs the survivin protein half-life suggests another mechanism by which ERK signaling may promote the survival and growth of pancreatic β-cells.

The EGF-dependent early increase in survivin protein is not significantly regulated at the level of transcription, as there is no significant EGF-dependent increase in *survivin *mRNA observed during these early time periods, nor is the *survivin *promoter activated. By contrast, experiments conducted in the presence of the protein synthesis inhibitor cyclohexamide support a post-translational mechanism of EGF-dependent regulation, with a decrease in protein degradation rate observed in the presence of EGF. In contrast to the 30 minute half-life of survivin in the absence of EGF, the survivin half-life is increased to 90 minutes in the presence of EGF.

The ubiquitin-proteosome pathway plays a central role in the regulation of multiple proteins involved in cellular homeostasis [45]. Many short-lived, key regulator proteins including the cyclins (cyclin A, B, D, E), cyclin kinase inhibitors CKI (p21, p27, p57), and transcription factors are regulated by this pathway [46]. Ubiquitin-proteasomes also regulate programmed proteolysis of pro- and anti-apoptotic proteins, including Bcl-2 family proteins and IAPs [46]. Previous work demonstrated that the ubiquitin-proteasome pathway regulates survivin degradation in a cell cycle-dependent manner [33]. Deubiquitination of survivin is also required for the proper targeting of survivin and its partner chromosomal passenger proteins to centromeres [34], enabling accurate cell division to take place. Our data suggest that EGF can inhibit ubiquitination of survivin in pancreatic β-cells, thereby prolonging its protein half-life.

EGF is currently in human clinical trials to treat insulin-deficient diabetes (Transition Therapeutics, Inc), with preliminary results showing a decrease in exogenous insulin requirements. It is unclear if this observed effect is due to improved β-cell function or to increases in β-cell proliferation of the remaining β-cells. Our results show that EGF enhances survivin stability through activation of the Raf-1/ERK pathway in the pancreatic β-cell lines MIN6 and INS-1 and support future work in exploring this pathway *in vivo*.

## Methods

### Cell culture

MIN6 (early passage, < 30) and INS-1 cells (line 832/13, passage < 25) were grown under proliferating conditions in DMEM/10% FBS. Cells were serum-deprived overnight prior to the addition of EGF (50 - 100 ng/ml) or IGF-1 (100 ng/ml) for 2 to 4 hours prior to adding glucose or insulin. To inhibit the EGF signaling pathways, cells were treated with the following compounds: LY 249002 (50 μM), UO126 (10 μM), SP600125 (20 μM) and AG490 (50 μM) (EMD Biosciences, San Diego CA) 30 minutes prior to EGF treatment then harvested for Western blot at the times indicated.

### Primary islet cell treatment

Pancreatic islets were isolated from 8-week old C57/Bl6 mice, as described previously [47]. The islets were separated by gradient centrifugation with Histopaque 1077 (Sigma) then handpicked under a dissection microscope and recovered in RPMI1640 media containing 10% FBS overnight. The following day, 100 to 500 islets were placed in 35 mm dishes containing RPMI1640 media without FBS. After overnight starvation, islets were treated for 2 h with EGF (100 ng/ml) then harvested for RNA. These experiments were approved by the Institutional Animal Care Board (IACUC) at Rhode Island Hospital.

### Western blotting and immunoprecipitation

Whole cell lysates were collected in RIPA buffer for Western blot analyses. 50 μg of protein per lane was separated on a 12-15% SDS-PAGE. Gels were transferred to nitrocellulose membranes then blotted with a rabbit anti-survivin antibody (Santa Cruz Biotechnology sc-10811, 1:1000) in 5% non-fat dry milk (NFDM), followed by anti-rabbit antibody (GE healthcare # NA934, 1:2000). Nuclear extracts from INS-1 cells were prepared according to the method of Schreiber et al.[48]. Total protein concentration was measured using BCA protein assay kit (Thermo Scientific) against a bovine serum albumin standard curve. Cells were collected, washed with PBS twice and pelleted by centrifugation. The cell pellet was resuspended in 50 μl cold buffer A (10 mM Tris pH 7.5; 10 mM KCl; 0.1 mM EGTA; 1% NP-40; 1 mM DTT; 1 mM PMSF; 1 complete Mini tablet) vortexed for 10 sec, then shaken on a rocker vigorously for 10-15 min. The lysate was centrifuged at 13000 RPM for 10-15 min. The supernatant containing cytoplasm was transferred to a fresh tube. The nuclear pellet was resuspended in 50 μl ice-cold buffer C (20 mM Tris pH 7.9; 400 mM NaCl; .5 mM EGTA; 1.5 mM MgCl2; 25% Glycerol; 1 mM DTT; 1 mM PMSF) and the tube vigorously rocked at 4°C for 15 min on a shaking platform. The nuclear extract was centrifuged for 10 min at 13000 RPM at 4°C and the supernatant (nuclear extract) transferred to a new tube. Nuclear or cytoplasmic fractions were resolved by SDS-PAGE. PVDF membranes were probed with mouse anti-survivin (Santa Cruz Biotechnology, sc-17779, 1:1000), rabbit anti-HDAC1(Affinity Bioreagents, #PA1-860, 1:5000) or mouse anti-Actin (Santa Cruz Biotechnology, sc-47778, 1:1000).

For the ubiquitin experiments, INS-1 cells were pretreated with MG132 (10 uM) for 3 h before being treated with EGF (100 ng/mL) for 1 h. Lysates were precleared with 15 μl of Protein A/G PLUS-Agarose (Santa Cruz Biotechnology, sc-2003) for 1 h prior to overnight incubation with 1 μg of either normal rabbit IgG (Santa Cruz Biotechnology, sc-2027) or rabbit polyclonal Survivin (Santa Cruz Biotechnology, sc-10811) primary antibody. 30 μl of Protein A/G Plus-Agarose beads were then added to form immunocomplexes and samples were shaken for 2 h. Samples were then spun at 3000 RPM for 5 min. Supernatant was discarded and Protein A/G Plus-Agarose beads were washed 4 times and then resuspended and boiled in a SDS loading buffer. All previous steps are performed at 4°C unless otherwise noted. Immunocomplexes were resolved by SDS-PAGE. PVDF membranes were probed with mouse anti-survivin (Santa Cruz Biotechnology, sc-17779) or rabbit anti-ubiquitin (Cell Signaling, #3933 S, 1:1000).

### Cyclohexamide treatment

INS-1 cells were starved overnight then treated with or without EGF (100 ng/ml) for a total of four hours. CTX (Calbiochem # 239765 100 mg/ml) was added 1.5 h prior to the completion of the four-hour EGF treatment.

### Analysis of mRNA expression

INS-1 and primary islet cells were harvested for RNA using the Qiagen RNAeasy kit (Qiagen Inc, Valencia CA). RNA was reverse transcribed into cDNA using Omniscript reverse transcriptase (Qiagen Inc, Valencia CA). qRT-PCR for *survivin *and *gapdh *were performed using the SYBR Green Master Mix kit from Applied Biosystems (Applied Biosystems Inc, Foster City, CA). Primers for total mouse *survivin *were as follows: Forward-Primer GATCTGGCAGCTGTACCTCA; Reverse-Primer ATTGACTGACGGGTAGTCTTTG. Primers for full-length rat *survivin *were as follows: Forward-Primer 5' CCGATCTGGCAGATGTACCT, Reverse-Primer 5' AGGGGAGTGCTTCCTATGCT. Survivin expression levels in the presence of EGF, relative to those in the absence of EGF were calculated using the ΔΔCT method in comparison to the housekeeping gene *GAPDH*. Experiments were performed in triplicate (INS-1 cells) or duplicate (primary islets).

### Luciferase assays

Promoter pGL2-enhancer constructs containing DNA varying in length from 400 to 6000 bp upstream of the *survivin *transcription start-site were a kind gift of Drs. Hatono and Tokuhisa [32]. The upstream DNA segments were removed from the pGL2-enhancer vector and ligated upstream of the luciferase reporter in the pGL4.10[luc2] (Promega Corp., Madison WI) vector without additional enhancer elements. Plasmids were transfected into MIN6 cells, serum deprived overnight then treated with EGF (100 ng/ml) for 2 h. Luciferase reporter activity was measured using the Promega Dual Glo kit (Promega Corp., Madison WI). Experiments were performed in triplicate.

### Immunofluorescence

INS-1 cells were grown to approximately 50% confluence in an 8 chambered glass slide, serum-starved and treated with EGF as above. Cells were fixed in 3.7% formaldehyde, 0.2% TritonX-100/PBS for 15 minutes at room temperature. Blocking was done in 1% BSA, 5% NGS/PBS for 1 hour. Primary antibody was rabbit anti-survivin (FL-142, Santa Cruz Biotechnology, CA). Secondary antibody was anti-rabbit IgG conjugated to Dylight 488 (ThermoFisher Scientific, Waltham MA). The slide was mounted with Prolong anti-fade reagent (Life Technologies, CA). Images were captured using a Nikon C1si Confocal microscope.

## Abbreviations

EGF: epidermal growth factor; IGF-1: insulin-like growth factor; CTX: cyclohexamide; IP: immunoprecipitation.

## Authors' contributions

HW-generated the data for figs. 1 and 3. KG-initiated the project, generated preliminary data, and wrote the preliminary draft. ZAC-generated data for figs. 2 and 4. DI-provided technical assistance. MPH-generated data for figs. 2 and 4. AK-performed the CTX experiments. CMB-conceived the project and helped with study design. KC-provided technical assistance. RAA-designed the study and wrote the manuscript. All authors read and approved the final manuscript.
